# Reducing Surface Wetting Proportion of Soils Irrigated by Subsurface Drip Irrigation Can Mitigate Soil N_2_O Emission

**DOI:** 10.3390/ijerph15122747

**Published:** 2018-12-05

**Authors:** Qi Wei, Junzeng Xu, Yawei Li, Linxian Liao, Boyi Liu, Guangqiu Jin, Fazli hameed

**Affiliations:** 1 State Key Laboratory of Hydrology-Water Resources and Hydraulic Engineering, Hohai University, Nanjing 210098, China; weiqi8855116@163.com (Q.W.); jingq02@126.com (G.J.); 2 College of Water Conservancy and Hydropower Engineering, Hohai University, Nanjing 210098, China; 3 College of Agricultural Engineering, Hohai University, Nanjing 210098, China; yaweizx@163.com (Y.L.); liaolinxian@hhu.edu.cn (L.L.); boyi1027ns@163.com (B.L.); fazlihameed@yahoo.com (F.h.)

**Keywords:** subsurface drip irrigation, partial wetting, nitrous oxide, sub-region, surface soil wetting proportions

## Abstract

To reveal the impact of soil moisture distributions on nitrous oxide (N_2_O) emissions from wet soils irrigated by sub-surface drip irrigation (SDI) with different surface soil wetting proportions, pot experiments were conducted, with surface irrigation (SI) as a control. Results indicated that irrigation triggered N_2_O pulsing effect in all SDI treatments, yet N_2_O values reduced with the decrease of surface soil wetting proportions of SDI irrigated soils, and the occurrence times were lagged. The peak N_2_O fluxes and the corresponding soil water filled pore space (WFPS), as well as the coefficients of determination (R^2^) of the exponential function between N_2_O fluxes and soil WFPS, decreased with the reduction of surface soil wetting proportions with SDI treatment, and from the central sub-region to the periphery sub-region. The pulse period contributed most to the reduction of N_2_O emissions in SDI compared to SI treatments and should be a key period for N_2_O emission mitigation. Over the whole experimental period, the area-weighted average cumulative N_2_O fluxes from SDI treatments were 82.3–157.3 mg N_2_O m^−2^ lower than those from SI treatment, with periphery sub-regions of R3 and R4 (radius of 19–27 cm and 28–36 cm from the emitter horizontally) contributing to more than 75.8% of the total N_2_O emission mitigation. These results suggest that reducing surface soil wetting proportions or the increments of topsoil WFPS for SDI irrigated soils is a promising strategy for N_2_O emission reduction.

## 1. Introduction

Nitrous oxide (N_2_O) is one of the most important atmospheric trace gases, with a global warming potential 298 times that of carbon dioxide over a 100-year scale [1,2,3]. It is also a key compound involved in the destruction of stratosphere ozone [4]. According to the Intergovernmental Panel on Climate Change (IPCC) fifth assessment report, agricultural ecosystems contribute 59.4% of the global anthropogenic N_2_O emissions, and the emissions from soils under natural vegetation account for 60% of natural source N_2_O emissions [2]. Thus, soil N_2_O emission has become a hot topic in the field of global greenhouse gas emissions [5,6].

Water management has been recognized as one of the key factors that triggers N_2_O emissions from soils [7,8,9]. Soil moisture controls the biotic and abiotic processes involved in the production, consumption and diffusion of N_2_O within the soil. Generally, denitrification rates increase rapidly when water filled pore space (WFPS) exceeds 60%, due to the decrease of oxygen supply, and predominates over nitrification in the production of N_2_O [10]. Yet, nitrification may contribute to a large proportion of soil N_2_O emissions when WFPS falls in the range of 30–70% [11]. 

Furthermore, soil WFPS is affected, both temporally and spatially, by the amount of irrigation water and its corresponding irrigation method, which inevitably affect the production and emission of N_2_O. Under surface irrigation (SI), where water is applied evenly to the soil surface, soil moisture is expected to be distributed homogeneously. However, for partial wetting irrigation, such as subsurface drip irrigation (SDI) or drip irrigation (DI), water is applied by a point source emitter at a low rate [12], which generally results in a non-uniform distribution of soil moisture in both horizontal and vertical directions [13,14]. The differences in soil moisture distribution might lead to changes in soil-related factors (such as soil aeration, the transformation of nitrogen, the distribution of soil NH_4_^+^ and NO_3_^−^, and soil microbial community composition), and thus in the production and emission of N_2_O [15,16]. It has been suggested in the literature that SDI might be a promising water management practice for mitigating soil N_2_O emissions [11,17,18]. Recently, N_2_O emissions from different sub-regions around the emitter were investigated under a specific wetting pattern, and it was found for the first time that N_2_O fluxes decreased gradually from the central sub-region to the periphery sub-region around the emitter [19]. For SDI, the soil wetting pattern will vary greatly due to the depth the emitter is set at or differences in the irrigation water volumes [20,21], which might lead to differences in the distribution of soil moisture, and hence N_2_O emissions. However, N_2_O emissions from SDI irrigated soils under different surface soil wetting proportions are rarely mentioned, and N_2_O emission responses to soil moisture among different SDI wetting patterns are still unclear. 

Thus, pot experiments were conducted on a silty clay soil with three surface soil wetting proportions produced by SDI. The objectives were to quantify N_2_O emissions from SDI irrigated soils under different surface soil wetting patterns, and to reveal the differences in N_2_O emission responses to soil moisture among SDI wetting patterns. 

## 2. Materials and Methods 

### 2.1. Soil Characteristics

Experiments were conducted on a silty clay soil from August to December 2014 at the State Key Laboratory of Hydrology-Water Resources and Hydraulic Engineering in Nanjing, China (32°03′ N, 118°46′ E). Air temperature during the experimental period ranged from 15.8 to 31.3 °C, and the precipitation was summed as 98 mm. Soil samples were collected in 2012 from a greenhouse after the tomato harvesting (*Solanum lycopersicum* L.) in the Vegetables Scientific Institute of Nanjing, China (31°56′ N, 118°37′ E). Soil organic matter, total nitrogen, and total phosphorus were 25.2, 1.4 and 0.04 g kg^−1^, respectively. Soil ammonium nitrogen and nitrate nitrogen were 8.8 and 34.2 mg kg^−1^, respectively and the soil pH_H2O_ was 6.2. Soil samples were air-dried, ground, sieved, homogenized, and finally packed into soil columns to the bulk densities of 1.25 and 1.33 g cm^−3^ for 0–10 cm and 10–50 cm layers, respectively, according to the local soil bulk densities. To avoid the influence of soil disturbance on soil N_2_O emissions, soil columns were left undisturbed for more than 60 days. During the experimental period, soil columns were placed outdoors in a completely randomized pattern and covered by an awning on rainy days only, to avoid the influence of rainfall.

### 2.2. Experimental Design

**Figure 1:**
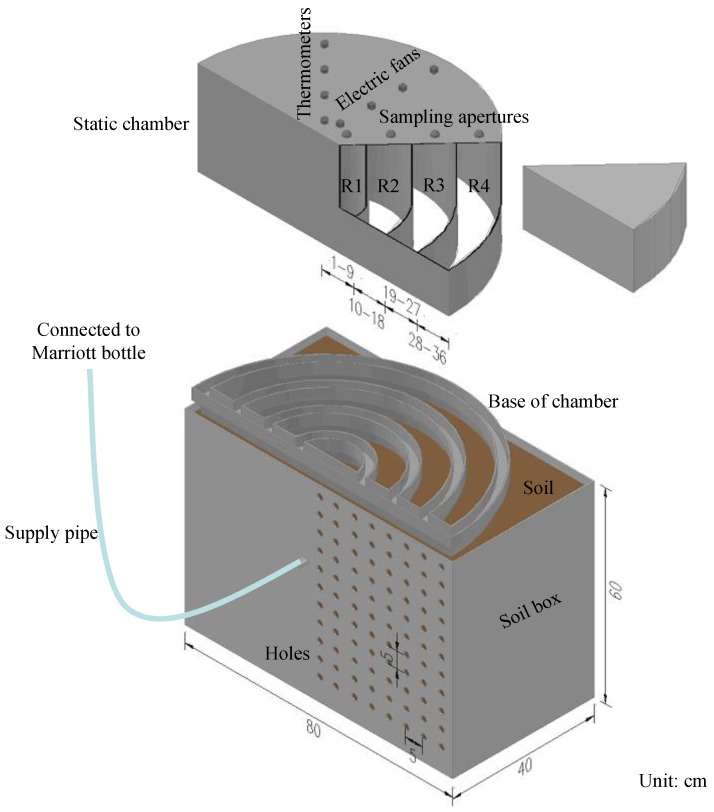
Diagram of sub-surface drip irrigation (SDI) box and the static chamber.

To investigate the effects of moisture distribution on N_2_O emissions from soils with different wetting patterns produced by SDI, three surface wetting proportions (a large, a little or almost no proportion of surface soil wetting, namely SDI–1, SDI–2, and SDI–3) were realized by placing SDI emitters at different depths (20, 25 and 30 cm) and supplying different amounts of water (Figure 1), with surface irrigation (SI) as a control. Experiments were set in 24 soil boxes (SI soil box: L × W × H = 40 × 40 × 60 cm; SDI soil box: L × W × H = 80 × 40 × 60 cm) with six replications (three for gas sampling and another three for soil sampling). For SI treatment, water was evenly applied to the soil surface to replenish 0–20 cm topsoil to field capacity. For SDI treatment, water was supplied from an SDI emitter connected to a Marriott bottle to form three specific soil partial wetting patterns (or surface soil wetting proportions). 

### 2.3. Irrigation Management and Soil Moisture Measurement

Soil columns were irrigated twice during the experimental period, at 10:00 h on 24 August and 9 October 2014. Soils in the SI, SDI–1, SDI–2, and SDI–3 boxes were irrigated with 7500, 3620, 2895 and 2500 mL of water, respectively, for the first irrigation, and with 7000, 3260, 2610 and 2200 mL of water, respectively, for the second irrigation. To map soil moisture distribution, soil samples were taken daily during the first 5 d and then at intervals of 3–5 d after each irrigation. For SI treatment, soil samples (approximately 3–5 g each) from depths of 0 to 24 cm in 4 cm increments were collected through holes (Inner diameter (I. D.) = 20 mm) located in one side of each soil box using a stainless steel sampler (I. D. = 18 mm). For SDI treatment, soil samples were collected in horizontal and vertical directions through holes (I. D. = 10 mm) located in one side of each soil box using a stainless steel sampler (Outer diameter (O. D.) = 8 mm), at 5 cm intervals from 0 to 55 cm layers vertically and 2.5 to 37.5 cm horizontally. Soil moisture content was determined gravimetrically by drying at 105 °C for 24 h, and WFPS was calculated as the percentage of soil volumetric moisture content (calculated by multiplying gravimetric soil moisture content by bulk density) to soil porosity, with total porosity calculated from the soil bulk density and particle density (2.65 g cm^−3^) [22]. 

### 2.4. Sampling and Analysis of N_2_O

Gas samples in four sub-regions (R1, R2, R3 and R4, with a radius of 1–9 cm, 10–18 cm, 19–27 cm and 28–36 cm, horizontally from the emitter, respectively) from each SDI soil column were collected using a static chamber (H = 40 cm, designed specially with four compartments, Figure 1) at 10-min intervals from 9:45 h–10:15 h. At the same time, gas samples from the SI soil column were collected using a cubic static chamber (L × W × H = 40 cm × 40 cm × 40 cm) with one compartment. The static chambers were made of 10 mm thickness polyvinyl chloride (PVC), and equipped with thermometers and electric fans at the top for measuring air temperature and mixing air inside. To minimize the impact of side heating, the chambers were wrapped with a layer of heat insulation material. Before gas sampling, the bases of the chambers were inserted into soil at a depth of 5 cm. 

N_2_O concentrations were analyzed using a gas chromatograph system (Agilent 7890B; Agilent Technologies Inc., Santa Clara, CA, USA) with an electron capture detector (ECD). Fluxes of N_2_O were calculated according to the linear increments of N_2_O concentration and the temperatures within the chambers [6]. Cumulative N_2_O emissions from each sub-region were calculated by integrating N_2_O fluxes along the sampling period. Area-weighted average N_2_O emissions from each SDI treatment were calculated based on the cumulative emissions from sub-regions R1–R4 with percentages (surface area proportion of each sub-region to the sum) of 7%, 19%, 31% and 43%, respectively.

### 2.5. Statistical Analysis

Statistical analyses were performed using SPSS19.0 (SPSS Inc., Chicago, IL, USA). Paired *t*-tests were performed to detect differences in N_2_O fluxes among SDI irrigated soils with different surface soil wetting proportions. Pearson’s correlation analyses were performed to investigate the relationships between soil moisture and N_2_O fluxes.

## 3. Results

### 3.1. Soil Moisture Distribution

**Figure 2:**
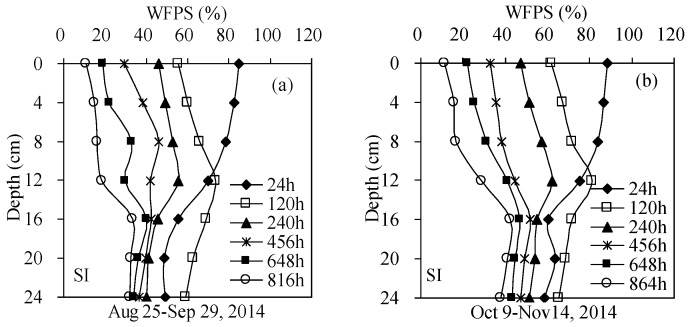
Soil moisture from surface irrigation (SI) treatment at different depths after the first (**a**) and second (**b**) irrigation.

**Figure 3:**
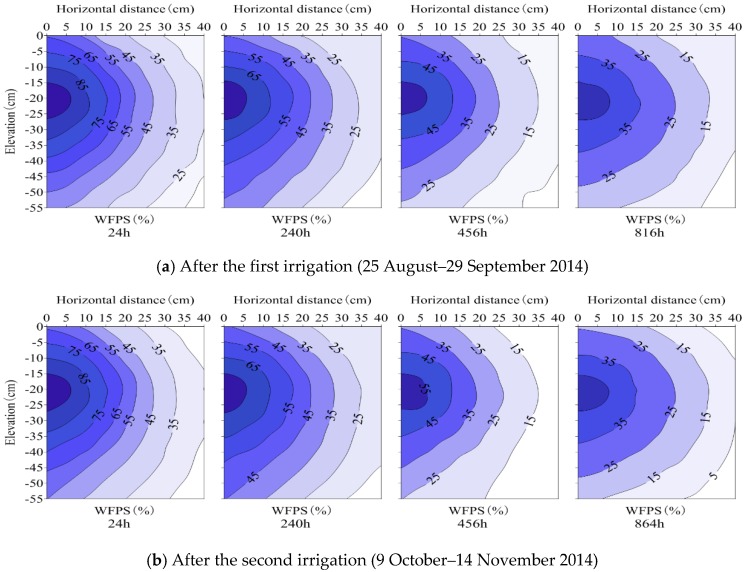
Soil moisture distribution in SDI–1 treatment (surface soil wetting proportion is 39.7%).

**Figure 4:**
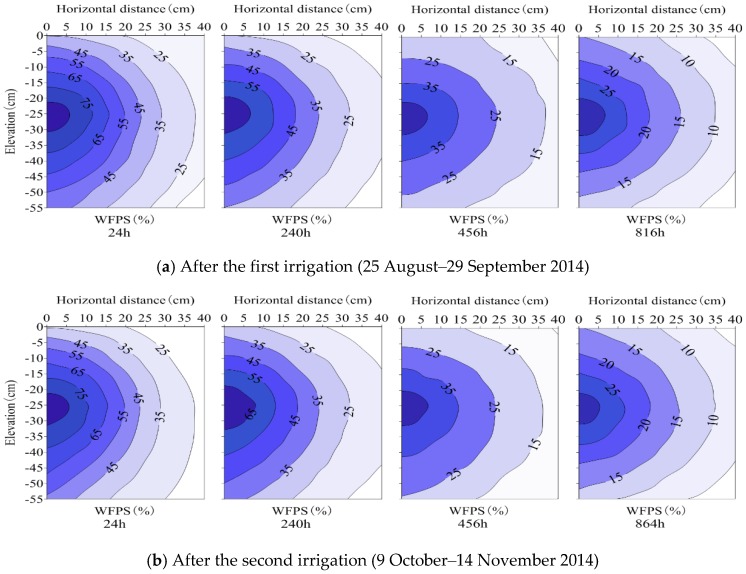
Soil moisture distribution in SDI–2 treatment (surface soil wetting proportion is 10.9%).

**Figure 5:**
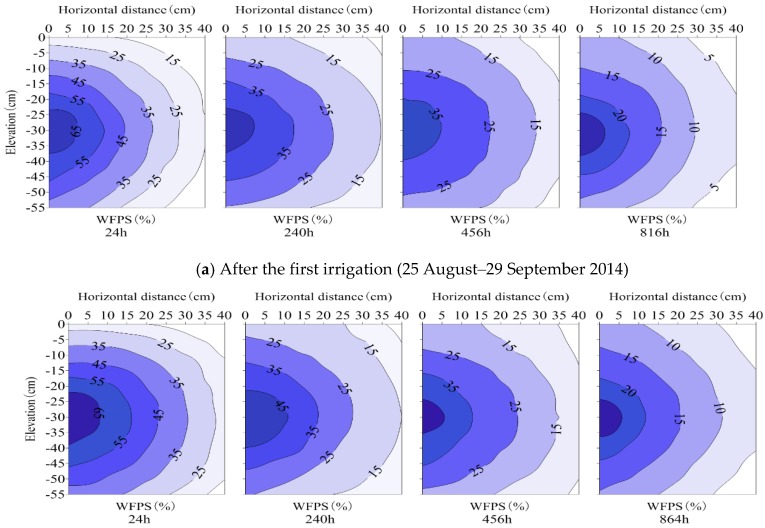
Soil moisture distribution in SDI–3 treatment (surface soil wetting proportion is 1.7%).

Soil moisture at 0–12 cm layers varied markedly in both the SI and SDI treatments (Figure 2, Figure 3, Figure 4 and Figure 5), due to the matric potential gradient and evaporation. For SI treatment, soil WFPS in the top 0–12 cm layers were within the ranges of 12.2%–81.2% and 12.6%–80.1% after the first (24–816 h) and second (24–864 h) irrigation, respectively. Soil moisture was much higher in the top layers than in the deep layers during the first 120 h after each irrigation. After that, soil moisture decreased significantly in the top layers and increased slightly in the deep layers (Figure 2). 

For SDI treatments, surface soil wetting proportions after the initial irrigation were 39.7% (SDI–1), 10.9% (SDI–2) and 1.7% (SDI–3), respectively. Soil moisture among the SDI wetting patterns showed a similar pattern, and soil wetting bodies were ellipsoid in shape with emitters at the center (Figure 3, Figure 4 and Figure 5). Soil moisture migrated gradually through the sub-regions, and the contour lines of soil moisture distributed in an approximate ball-crown body which were more or less parallel to each other in the vertical profile. Soil moisture was distributed non-uniformly in both horizontal and vertical directions and was redistributed after irrigation events. For each specific SDI treatment, soil moisture decreased gradually in a horizontal direction with increasing radial distance to the emitter. Among SDI treatments, the soil area-weighted average WFPS reduced from SDI–1 to SDI–3. 

For treatment SDI–1, the soil WFPS was above 60% in sub-regions near the emitter during the first 48–72 h after water was applied, whereas at the wetting front (the interface between wet and dry areas) it was less than 40%. At 24 h after the first irrigation, the average WFPS in 0–20 cm of soil reduced horizontally from 82.3% at 2.5 cm from the drip emitter, to 25.4% at 37.5 cm from the drip emitter. Then, soil moisture in different sub-regions decreased with elapsed time, and reduced to 20.8%–63.0%, 11.5%–46.6% and 9.2%–39.8% at 240 h, 456 h and 816 h after the first irrigation, respectively. Soil moisture among sub-regions after the second irrigation showed a similar pattern to that after the first irrigation, although the area-weighted average (0–20 cm) WFPS was 0.2%–3.5% higher. Compared to SDI–1, soil moisture for SDI–2 and SDI–3 treatments showed a similar pattern, although the area-weighted average WFPS was reduced by 3.4–16.7% and 4.0%–15.4% after the first irrigation, and by 4.7%–21.3% and 4.2%–18.5% after the second irrigation, respectively (Figure 3, Figure 4 and Figure 5).

### 3.2. N_2_O Emission

**Figure 6:**
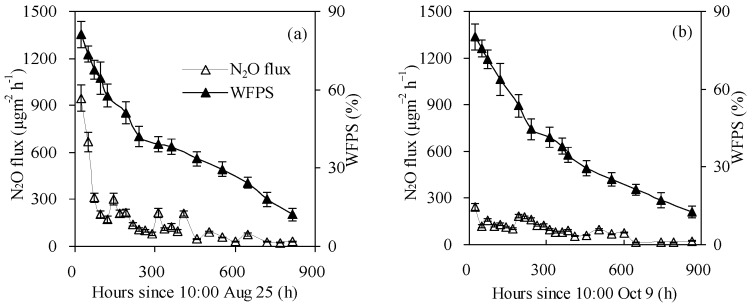
N_2_O emission fluxes from SI treatment after the first (**a**) and second (**b**) irrigation.

N_2_O fluxes from SI treatment were maintained at a high level at the beginning (0–48 h and 0–24 h after the first and second irrigation, respectively) of the experimental period; following this, N_2_O fluxes reduced with the decrease of soil average (0–12 cm) WFPS (Figure 6). The maximum N_2_O fluxes of 946.4 and 243.1 μg N_2_O m^−2^ h^−1^ were observed at 24 h after both irrigations, and the corresponding soil average (0–12 cm) WFPS were within the ranges of 75.6%–85.1% and 74.5%–84.3%. Irrigation events resulted in pulse N_2_O emissions from SI irrigated soil. In addition, the peak N_2_O flux after the second irrigation was seven times greater than that at 816 h after the first irrigation (or prior to the second irrigation). 

**Figure 7:**
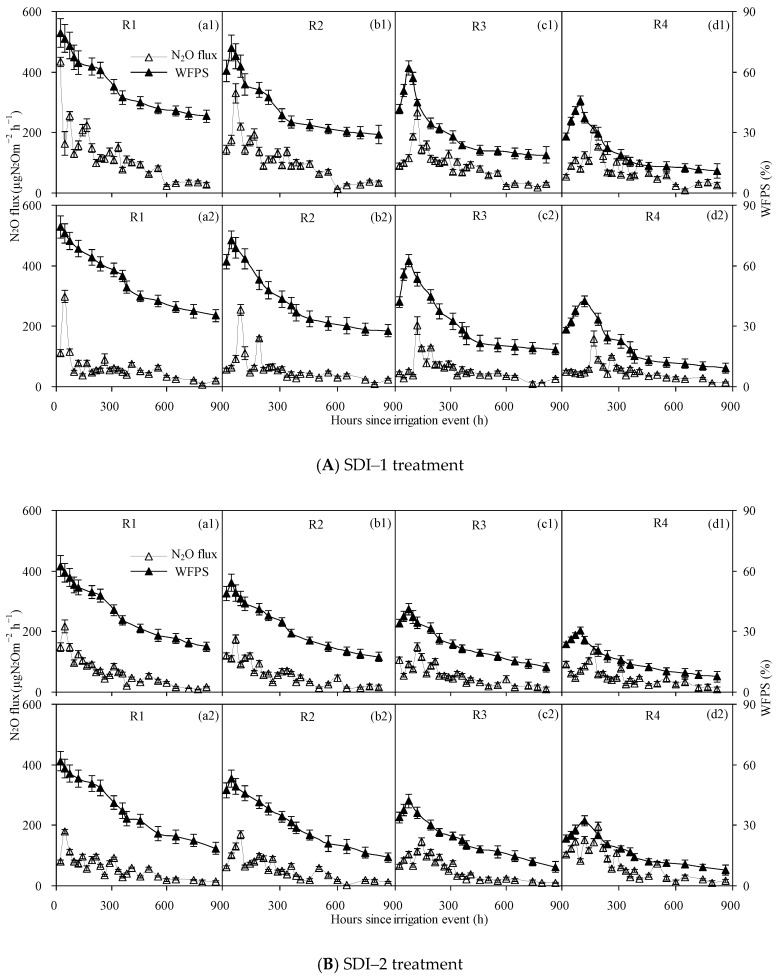

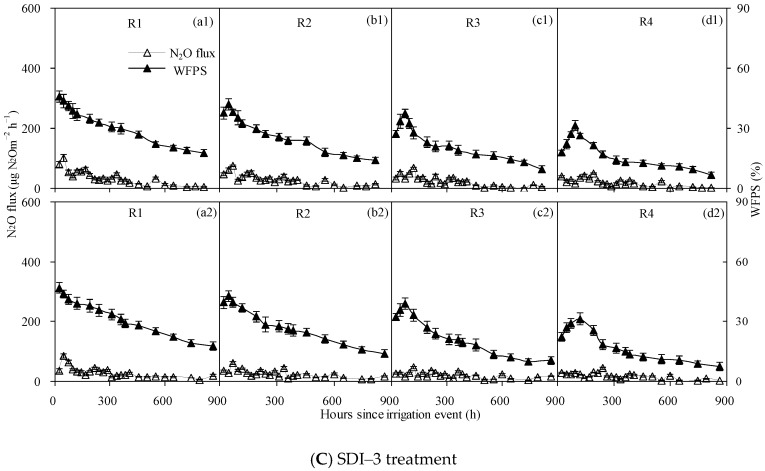
N_2_O emission fluxes in different sub-regions (**A**–**C**) from SDI treatments after the first irrigation (**a1**–**d1**) and the second irrigation (**a2**–**d2**).

N_2_O fluxes from SDI treatments differed slightly among sub-regions and among surface soil wetting patterns (Figure 7) and were all significantly lower than those from SI treatment (Figure 6). For SDI–1 treatment, N_2_O fluxes in sub-region R1 increased rapidly during the first 48 h after both irrigations, with peaks of 433.7 and 298.5 μg N_2_O m^−2^ h^−1^ observed at 24 h and 48 h, respectively, and corresponding soil average (0–20 cm) WFPS of 76.1% and 79.5% (Figure 7A). Then, N_2_O fluxes gradually declined with the decrease in soil moisture content and reduced to 24.6 and 8.1 μg N_2_O m^−2^ h^−1^ at 816 and 864 h after the first and second irrigation, respectively. Compared with sub-region R1, soil N_2_O emissions from sub-regions R2, R3 and R4 showed a similar pattern, although the peak times were lagged by 48 h, 72–96 h and 120–144 h, and peak values were decreased by 14.8%–23.8%, 32.3%–38.4% and 46.9%–51.2%, respectively. Furthermore, the peak N_2_O fluxes in sub-region R1 occurred almost simultaneously with the maximum soil WFPS, while in sub-regions R2–R4, peak N_2_O fluxes occurred 24–72 h later than the corresponding maximum soil WFPS. The second watering–drying cycle also triggered large amounts of N_2_O emissions which were observed soon after the soil irrigation event, but peak N_2_O emissions occurred 0–24 h later and the peak values were 23.1%–31.2% lower compared to those after the first irrigation. Simultaneously, the intervals between the peak times of N_2_O emissions and the maximum soil WFPS were longer after the second irrigation than after the first irrigation. Over the two experimental periods, N_2_O fluxes were significantly higher in sub-region R1 than in sub-regions R2–R4 (*p* < 0.05), with fluxes in sub-region R4 having the lowest. Also, soil WFPS in 0–20 cm layers corresponding to the peak N_2_O fluxes decreased from sub-region R1 to sub-region R4. 

N_2_O fluxes from SDI–2 and SDI–3 treatments varied in patterns similar to those from the SDI–1 treatment, although the peak times lagged by 0–24 h and 24–72 h, and peak values decreased by 17.4%–49.9% and 71.5%–77.0% (*p* < 0.05), respectively (Figure 7B,C). Meanwhile, the intervals between the occurrence times of peak N_2_O emissions and the maximum soil WFPS were longer in SDI–2 (24–72 h) and SDI–3 (24–120 h) treatments than in SDI–1 (0–72 h) treatment.

Generally, N_2_O emissions from SDI irrigated soils were positively correlated with surface soil wetting proportions, and these fluxes were reduced from SDI–1 to SDI–3 treatments. Within a specific surface soil wetting pattern of SDI irrigated soil, N_2_O fluxes decreased from the central sub-region to the periphery sub-region and the fluxes were significantly lower than those from SI irrigated soil, reducing on average by 33.5%–54.5%, 54.4%–64.7% and 77.3%–86.7% for SDI–1, SDI–2 and SDI–3 treatments, respectively. Among sub-regions, the differences in N_2_O fluxes were more marked during the pulse period than during the post-pulse period. In particular, soil average N_2_O fluxes from sub-region R4 were significantly lower than those from sub-region R1 during pulse periods (0–240 h, 0–192 h and 0–240 h for SDI–1, SDI–2 and SDI–3 treatments, respectively) after the first irrigation (*p* < 0.05), yet no significant differences in N_2_O emissions were observed among sub-regions during the post-pulse period after the second irrigation. 

### 3.3. Reduction in N_2_O Emissions of SDI Soils 

**Figure 8:**
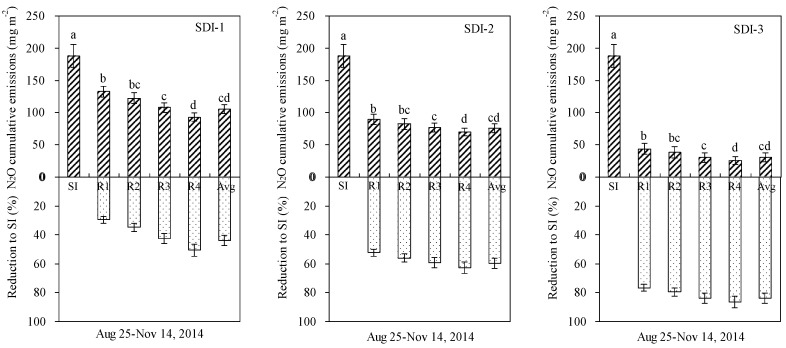
Cumulative N_2_O emissions in different sub-regions of SDI treatments and their reduction compared to SI treatment. ‘Avg’ represents area-weighted average value. Different letters on each bar represent significant differences in N_2_O cumulative emissions at *p* = 0.05 level.

Cumulative N_2_O emissions in all sub-regions from SDI–1, SDI–2 and SDI–3 treatments were within the ranges of 92.9–132.6, 70.0–89.6 and 25.5–43.7 mg N_2_O m^−2^, respectively, during two drying–wetting cycles, which were 29.4%–50.6%, 52.3%–62.8% and 76.9%–86.4% lower, respectively, than those from the SI treatment (Figure 8). The soil area-weighted average N_2_O fluxes decreased by 82.3, 112.3 and 157.3 mg N_2_O m^−2^, respectively, compared to SI treatment (187.8 mg N_2_O m^−2^), with differences significant at the *p* = 0.05 level. Moreover, cumulative N_2_O fluxes in sub-region R1 were significantly higher than those in sub-region R4 of all SDI treatments (*p* < 0.05), but no significant differences were observed between sub-regions R2 and R3 (Figure 8).

Compared to SI, SDI decreased cumulative N_2_O emissions by 40.2%–63.0% (SDI–1), 58.6%–67.7% (SDI–2) and 77.6%–85.4% (SDI–3) in the pulse period, and by 103.1%–221.8%, 19.4%–38.2% and 3.0%–7.2% in the post-pulse period, respectively (Table 1). Among SDI treatments, the reduction rates of N_2_O emission were in the sequence of, SDI–1 < SDI–2 < SDI–3, while rates of N_2_O emission reductions between the pulse period and the whole period showed the contrary, with area-weighted average N_2_O emissions reduction summing to 55.2% (SDI–1), 64.4% (SDI–2) and 85.4% (SDI–3) in the pulse period, and 43.8%, 59.8% and 83.7% during the two experimental periods, respectively. These results suggested that the pulse period contributed most to the reduction of N_2_O emissions between the SDI and SI treatments and should be a key period for N_2_O emission mitigation.

During two drying-wetting cycles, cumulative N_2_O emissions (calculated as cumulative N_2_O emissions along the sampling period from each specific sub-region) from all sub-regions were within ranges of 1.7–7.5, 1.1–5.6 and 0.5–2.1 mg for SDI–1, SDI–2 and SDI–3 treatments, respectively, and reduced from the central sub-region to the periphery sub-region for each specific SDI wetting pattern (Table 2). Compared with N_2_O emissions from SI treatment, cumulative N_2_O emissions from SDI treatments were decreased by 0.7–7.6, 1.2–9.5 and 1.8–13.0 mg. For each specific SDI treatment, periphery sub-regions of R3 and R4 contributed to more than 75.8% of the total N_2_O emission mitigation. 

### 3.4. Relationship between N_2_O Emissions and Soil WFPS 

Correlation analysis indicated that N_2_O fluxes from SDI irrigated soils increased exponentially with the increase of soil WFPS (Table 3). The coefficients of determination (R^2^) of these exponential functions were within the ranges of 0.334–0.465, 0.708–0.864 and 0.671–0.723 for SDI–1, SDI–2 and SDI–3 treatments, with values in the sub-region R2 from SDI–2 treatment slightly higher than those from SDI–1 and SDI–3 treatments. For each specific SDI wetting pattern, the R^2^ decreased from central sub-region to periphery sub-region.

## 4. Discussion

Previous research showed that pulse N_2_O emissions occurred immediately following heavy rainfall or irrigation, ranged from a few to 18,000 mg N_2_O m^−2^ h^−1^ [8,13,23] and contributed to between 34.3% and 98.7% of the cumulative N_2_O emissions [23,24]. The present study confirmed short-term pulse N_2_O emissions from SDI irrigated soils with different surface soil wetting proportions. The magnitudes and the contributions (to the total emissions) of peak N_2_O emissions found were consistent with the previous research. This was possibly because microbiological activity was very weak, and many bacteria and fungi died when the soil was dry which resulted in increasing concentrations of labile N and C. The addition of water (rainfall or irrigation) stimulated nitrification and de-nitrification processes and therefore increased N_2_O emissions [17,25,26]. These results suggest that the ‘pulsing effect’ can be attributed to the increased and variable fluxes of N_2_O caused by irrigation events.

Soil moisture is an important factor in triggering N_2_O emissions from soils [7,8,9], and its content significantly affected the magnitude of N_2_O fluxes [11,17,27]. Some researchers have indicated that peak N_2_O emissions were generally observed when the WFPS fell in the range of 45%–90% [8,28,29,30,31]. However, in the present research, soil WFPS corresponding to peak N_2_O fluxes were mainly within the ranges of 22%–79% (SDI–1), 17%–59% (SDI–2) and 13%–44% (SDI–3) (Figure 7), which were slightly lower than the results reported by previous research. This might be due to the fact that the processes involved in the production and consumption of soil N_2_O are very complex. These processes are simultaneously affected by multiple factors other than soil moisture such as soil texture, soil microorganisms and soil physicochemical properties, [8,13,28,32,33]. 

Generally, soil moisture in sub-region R1 decreased gradually over time, whereas in sub-regions R2–R4 it initially increased for a short period before it decreased. In particular, soil moisture in sub-regions R3 and R4 varied with a lag in the temporal process and a smaller variation amplitude than those in sub-regions R1 and R2. Consequently, the magnitude of pulse N_2_O emissions were smaller and the occurrence times were later in sub-regions R3 and R4. This phenomenon may be attributed to the following reasons: Firstly, soil moisture (10%–80% WFPS) under partial wetting irrigation (where insufficient water was supplied) was generally maintained at an appropriate range for N_2_O emissions, and N_2_O fluxes generally increased with the increase of moisture content within the moisture range [8,30]. Soil moisture decreased from the central sub-region to the periphery sub-region, and thereby soil N_2_O emissions reduced sequentially; secondly, the soil with a high water content moved downward from sub-region R1 to R4 (Figure 3, Figure 4 and Figure 5), weakening the N_2_O emission response to the corresponding soil moisture. These two reasons may account for the relationships between the magnitudes and occurrence times of peak N_2_O emissions among the sub-regions. Meanwhile, it is implied that reducing area-weighted average N_2_O emissions by allocating sub-surface R1–R4 SDI irrigated soils is a potential measure for N_2_O emission mitigation.

Additionally, some research addressing the relationships between N_2_O fluxes and soil WFPS from the perspective of temporal variation, showed that soil N_2_O fluxes increased linearly or exponentially with the increase of soil WFPS [8,30,31]. In the current research, correlational analyses indicated that N_2_O fluxes increased exponentially with the increase of soil WFPS in all SDI treatments, although the WFPS of these soils were slightly lower than some of the results reported in the literature [8,28,29,30,31]. The coefficients of determination (R^2^) of the exponential functions, as well as the average slopes, were in the sequence of SDI–2 > SDI–3 > SDI–1 and decreased gradually from the central sub-region to the periphery sub-region. These results suggest that the degree of impact of soil moisture on N_2_O emissions might be slightly different either among SDI wetting patterns, or among sub-regions.

**Figure 9:**
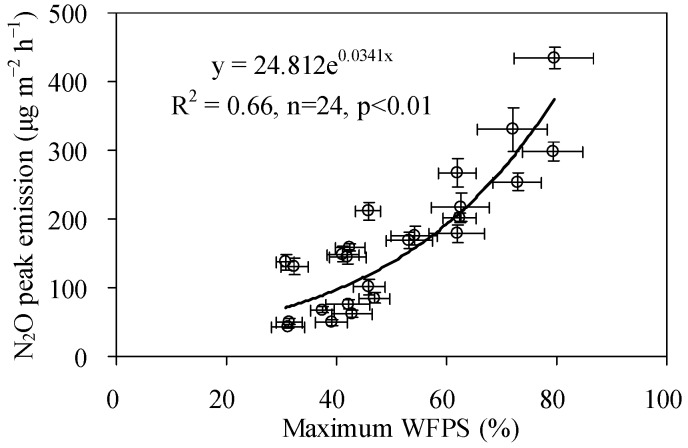
Relations between peak N_2_O fluxes and the maximum WFPS in SDI treatments.

In terms of spatial variation, peak N_2_O fluxes in different sub-regions from all SDI irrigated soils were found to increase exponentially with the increase of the maximum soil WFPS (N_2_O peak flux = 24.8 e^3.414 WFPS(Max)^, R^2^ = 0.663, *p* < 0.05, Figure 9). Thus, it can be inferred that the magnitudes of peak N_2_O fluxes are determined by the corresponding maximum WFPS values, even if the corresponding soil moisture level is lower than the previously reported optimal soil moisture level (45%–90% WFPS) for peak N_2_O emissions. This indicated that reducing the increment amplitude of soil WFPS in the topsoil of each sub-region was helpful for decreasing pulse N_2_O emissions. In addition, the decreased magnitude of peak emissions would be the final determinant of the total reduction potential of N_2_O emissions, as the reduction of N_2_O emissions in the pulse period contributed a larger proportion of the total N_2_O emission reduction (Table 1). 

Irrigation management is an important practice that determines soil moisture distribution characteristics, and therefore N_2_O emissions. With the development of water-saving irrigation technology, partial wetting irrigation methods (such as drip irrigation and subsurface drip irrigation) are applied widely to cope with water scarcity. For example, Sánchez-Martín et al. [11,17] investigated the influence of drip irrigation on N_2_O emissions from horticultural soil and proposed that the drip irrigation method can be used to save water and mitigate N_2_O emissions. Kallenbach et al. [13] demonstrated that the average N_2_O flux under SDI, which was calculated as average of N_2_O fluxes from three different locations (the plant line, shoulder of the soil bed, and furrow), was reduced by 50% compared to furrow irrigation. However, they did not consider the differences in N_2_O fluxes among different locations around the drip emitter. The current research tried to quantify N_2_O emissions from sub-regions around the emitter of soil irrigated by SDI with different surface soil wetting proportions. The results indicated that SDI resulted in decreased N_2_O emission from soils when compared to SI. Furthermore, N_2_O fluxes from SDI-irrigated soils were generally reduced with a decrease in surface soil wetting proportion, and from the central sub-region to the periphery sub-region. To reduce N_2_O emissions, it is better to reduce the surface soil wetting proportion and moisture level in topsoil in the sub-regions of SDI irrigated soils. Thus, an ideal or optimal soil wetting pattern should be designed by manipulating SDI technique parameters (such as emitter depths, irrigation water volume), with soil moisture high enough to meet crop water requirements as the prerequisite. Balancing the goal of decreased N_2_O emissions while properly meeting crop water requirements is another issue we should address in future research. It can be concluded that SDI is a prospective irrigation method for N_2_O emission reduction if it is designed properly, although it accounts for a very small proportion of the irrigation area in China, or even globally (no more than 0.6%) [34]. Considering that SDI has many advantages (such as saving water and fertilizer, enhancing crop yield and quality, improving labor productivity etc.), it should have great prospects in the future. Also, the current results imply that the wide application of SDI will play a positive role in reducing N_2_O emissions.

Other than soil moisture, nitrogen (N) fertilizer is another important factor that affected N_2_O emissions [35,36]. The impacts of moisture distribution patterns on N_2_O emissions should also be examined in field experiments under the conditions including crop cultivation and N fertilization management in the future. These results will be helpful for understanding the influence and mechanisms of water and nitrogen dynamics on crop growth and N_2_O emissions under SDI irrigation, and for guiding water and fertilizer management practices in agricultural production to realize N_2_O emission mitigation. 

## 5. Conclusions

N_2_O emissions from SDI irrigated soils with different surface soil wetting proportions were investigated in the present research. Results indicated that surface soil wetting patterns caused by SDI significantly affected soil N_2_O emissions. The wetting process among sub-regions under all SDI wetting patterns resulted in pulse N_2_O effects; however the magnitudes and occurrence times of pulse N_2_O fluxes were reduced and lagged with the decrease of surface soil wetting proportions. Generally, peak N_2_O fluxes from SDI irrigated soils increased exponentially with the increase of the maximum WFPS, and the magnitudes of pulse N_2_O fluxes were positively correlated with the match between the degree of soil WFPS and the optimal soil moisture level for peak N_2_O emissions. Compared to SI, N_2_O emissions from SDI irrigated soils reduced from SDI–1 (surface soil wetting proportion is 39.7%) to SDI–3 soil (surface soil wetting proportion is 1.7%). Furthermore, within a specific SDI wetting pattern, N_2_O emissions reduced from central sub-regions to periphery sub-regions. The pulse period contributed most to the reduction of N_2_O emissions between SI and SDI treatments and should be a key period for N_2_O emission mitigation. These results suggest that reducing the increment of topsoil WFPS or surface soil wetting proportions is a promising strategy for N_2_O emission reduction. However, further research should address more typical wetting patterns in circumstances in which the soil moisture is high enough to meet crop water requirement as the prerequisite, to explore an ideal or optimal soil wetting pattern by manipulating SDI technique parameters.

## Figures and Tables

**Table 1:** Reduction in N_2_O emissions from different sub-regions of SDI treatments at different observation periods.

Treatment (n = 3)	Sub-Region	Reduction in N_2_O Emission of SDI Treatment (%)			
		Observation Period			P/W
		Pulse Period	Post-Pulse Period	Whole Observation Period	
SDI–1	R1 (1–9 cm)	40.2	12.5	29.4	136.6
	R2 (10–18 cm)	43.9	20.5	34.8	126.1
	R3 (19–27 cm)	53.5	25.0	42.5	126.1
	R4 (28–36 cm)	63.0	31.0	50.6	124.6
	Avg	55.2	26.2	43.8	125.9
SDI–2	R1 (1–9 cm)	58.6	42.4	52.3	112.0
	R2 (10–18 cm)	60.3	49.6	56.1	107.4
	R3 (19–27 cm)	63.1	52.9	59.1	106.7
	R4 (28–36 cm)	67.7	55.0	62.8	107.8
	Avg	64.4	52.7	59.8	107.7
SDI–3	R1 (1–9 cm)	77.6	75.4	76.7	101.2
	R2 (10–18 cm)	82.3	76.7	79.6	103.4
	R3 (19–27 cm)	85.4	81.4	83.8	101.8
	R4 (28–36 cm)	87.6	84.6	86.4	101.3
	Avg	85.4	81.4	83.7	102.0

R1–R4 represent different sub-regions in the horizontal direction of SDI irrigated soils; ‘Avg’ represents weighted average value of different sub-regions of SDI treatment. P/W, means the ratio in N_2_O reduction rate in the pulse period to the whole observation period.

**Table 2:** Contribution to N_2_O emission reduction from different sub-regions of SDI treatments.

Treatment (n = 3)	Sub-Region	Region Area (cm^2^)	Cumulative N_2_O Emission (mg)	Reduction in N_2_O Emission (mg)	Contribution to Total N_2_O Reduction (%)
SDI–1	R1	125.6	1.7	0.7	4.6
	R2	351.7	4.3	2.3	15.1
	R3	577.8	6.2	4.6	30.2
	R4	803.8	7.5	7.6	50.1
	Total	1858.9	19.7	15.2	100.0
SDI–2	R1	125.6	1.1	1.2	5.9
	R2	351.7	2.9	3.7	17.8
	R3	577.8	4.4	6.4	30.8
	R4	803.8	5.6	9.5	45.5
	Total	1858.9	14.1	20.8	100.0
SDI–3	R1	125.6	0.5	1.8	6.2
	R2	351.7	1.3	5.3	18.0
	R3	577.8	1.8	9.1	31.1
	R4	803.8	2.1	13.0	44.7
	Total	1858.9	5.7	29.2	100.0

R1–R4 represent different sub-regions in the horizontal direction of SDI irrigated soils. Totals represent the summed value of different sub-regions of SDI treatment. Cumulative N_2_O emission was calculated during the incubation period for each specific sub-region. Reduction in N_2_O emission was calculated by subtracting the cumulative N_2_O emission of each sub-region from that for SI treatment in the same area.

**Table 3:** Relations between N_2_O fluxes and water filled pore space (WFPS) in SDI irrigated soils with different surface soil wetting patterns.

Treatment	Sub-Region	Relation	Coefficient of Determination (R^2^)	Significance
SDI–1	R1 (1–9 cm)	y = 6.265e^4.451x^	0.671	*p* < 0.05
	R2 (10–18 cm)	y = 14.622e^3.443x^	0.534	*p* < 0.05
	R3 (19–27 cm)	y = 23.651e^2.815x^	0.334	*p* < 0.05
	R4 (28–36 cm)	y = 21.963e^3.464x^	0.423	*p* < 0.05
	Overall	y = 23.010e^2.554x^	0.433	*p* < 0.05
SDI–2	R1 (1–9 cm)	y = 5.551e^5.517x^	0.864	*p* < 0.05
	R2 (10–18 cm)	y = 4.728e^6.573x^	0.733	*p* < 0.05
	R3 (19–27 cm)	y = 6.215e^7.618x^	0.807	*p* < 0.05
	R4 (28–36 cm)	y = 9.283e^8.134x^	0.708	*p* < 0.05
	Overall	y = 11.985e^4.462x^	0.561	*p* < 0.05
SDI–3	R1 (1–9 cm)	y = 2.639e^7.221x^	0.723	*p* < 0.05
	R2 (10–18 cm)	y = 3.763e^6.328x^	0.557	*p* < 0.05
	R3 (19–27 cm)	y = 3.126e^7.486x^	0.465	*p* < 0.05
	R4 (28–36 cm)	y = 3.107e^8.563x^	0.494	*p* < 0.05
	Overall	y = 3.799e^6.493x^	0.556	*p* < 0.05
